# Clinical correlation between premature ovarian failure and a chromosomal anomaly in a 22-year-old Caucasian woman: a case report

**DOI:** 10.1186/1752-1947-6-368

**Published:** 2012-10-29

**Authors:** Domenico Dell’Edera, Andrea Tinelli, Oronzo Capozzi, Annunziata Anna Epifania, Antonio Malvasi, Dominga Lofrese, Elena Pacella, Giusi Natalia Milazzo, Eleonora Mazzone, Manuela Leo, Mariano Rocchi

**Affiliations:** 1 Unit of Cytogenetic and Molecular Genetics, Madonna delle Grazie Hospital, Matera, 75100, Italy; 2 Obstetrics and Gynecology Department, V. Fazzi Hospital, Lecce, Italy; 3 Department of Biology, University of Bari, Bari, Italy; 4 Unit of Clinical Chemistry, Madonna delle Grazie Hospital, Matera, Italy; 5 Obstetrics and Gynecology Department, Santa Maria Hospital, Bari, Italy; 6 Department of Ophthalmology, Sapienza University, Rome, Italy; 7 S'Andrea Hosptital, Sapienza University, Rome, Italy

## Abstract

**Introduction:**

Premature ovarian failure is defined as the cessation of ovarian activity before the age of 40 years. It is biochemically characterized by low levels of gonadal hormones (estrogens and inhibins) and high levels of gonadotropins (luteinizing hormone and follicle-stimulating hormone).

**Case presentation:**

Our patient, a 22-year-old Caucasian woman under evaluation for infertility, had experienced secondary amenorrhea from the age of 18. No positive family history was noted regarding premature menopause. An examination of our patient’s karyotype showed the presence of a reciprocal translocation, apparently balanced, which had the X chromosome long arm (q13) and the 14 chromosome short arm (p12) with consequent karyotype: 46, X, t(X; 14)(q13;p12).

**Conclusions:**

Our study has underlined that karyotyping is one of the fundamental investigations in the evaluation of amenorrhea. It highlighted a genetic etiology, in the form of a chromosomal abnormality, as the causal factor in amenorrhea.

## Introduction

Premature ovarian failure (POF) is defined by the cessation of ovarian activity before the age of 40 years [1]. This condition is biochemically characterized by low levels of gonadal hormones (estrogens and inhibins) and high levels of gonadotropins (luteinizing hormone (LH) and follicle-stimulating hormone (FSH)) [2].

POF is considered idiopathic [3] in two thirds of cases, with the patient having a normal karyotype; in the remaining third of cases, it is secondary to genetic anomalies [4], autoimmune pathologies [5], pharmacological therapies [6], radiotherapy, or surgical oophorectomy.

In the absence of surgical oophorectomy, chemotherapy or pelvic radiation, POF encompasses a heterogeneous spectrum of conditions through two major mechanisms, follicle dysfunction and follicle depletion [7]. Although there are many other reasons for ovarian failure, genetic or chromosomal causes are the most important as their presence affects subsequent management.

Our study emphasizes that karyotyping is one of the fundamental investigations in the evaluation of amenorrhea. It highlights a genetic etiology, in the form of a chromosomal abnormality, as the causal factor in amenorrhea.

## Case presentation

Our patient, a 22-year-old Caucasian woman under evaluation for infertility, had experienced secondary amenorrhea from the age of 18 years. She had received hormonal replacement for the past two years, which resulted in cyclical bleeding, but she remained anovulatory. No positive family history was noted regarding premature menopause.

Pelvic ultrasonography showed the presence of an anteflexed uterus, with a normal profile, echostructure and dimensions. Her endometrium had a normal echographic aspect. Both her right and left ovary were normal with respect to dimension and form, without any liquid effusion. A hysterosalpingogram confirmed the normal uterus-tubal anatomy.

Magnetic resonance imaging of her encephalon and hypophysis using paramagnetic contrast showed that her sellar cavity had regular morphology and dimensions, without any structural alterations. The adenohypophysis was devoid of alterations. Her hypophyseal peduncle oriented normally. Serum anti-ovarian and anti-adrenal antibodies were absent. As it is possible to see from the Table 1, our patient had high levels of gonadotropins (LH: 41.17IU/L and FSH: 79.90IU/L; hypergonadotropic amenorrhea) [2]. Her thyroid-stimulating hormone, free tri-iodothyronine and free thyroxin hormone levels were normal, while the levels of anti-thyroid peroxidase antibodies and anti-thyroglobulin antibodies were very high.

**Table 1:** Hormonal assays

**Hormonal evaluation**	**Result**	**Normal range**
Thyroid-stimulating hormone (mIU/mL)	3.01	0.40 to 4.50
Free tri-iodothyronine (pg/mL)	4.21	2.30 to 5.10
Free thyroxin (ng/dL)	1.32	0.80 to 2.00
Thyroglobulin (ng/mL)	3.2^a^	<25
Anti-thyroid peroxidase antibodies (IU/mL)	172^a^	<30
Anti- thyroglobulin antibodies (IU/mL)	488^a^	<50
Parathyroid hormone(ng/mL)	32.42	9 to 72
Follicle-stimulating hormone (IU/L)	79.90^a^	<30
Luteinizing hormone (IU/L)	41.17^a^	<9
17-beta-estradiol (pg/mL)	9	<10
Prolactin (ng/mL)	6.2	<20
Testosterone (ng/mL)	0.22	0.1 to 1.0
Testosterone-free (pg/mL)	1.4	1.1 to 3.1

^a^Outside of normal range.

**Figure 1:**
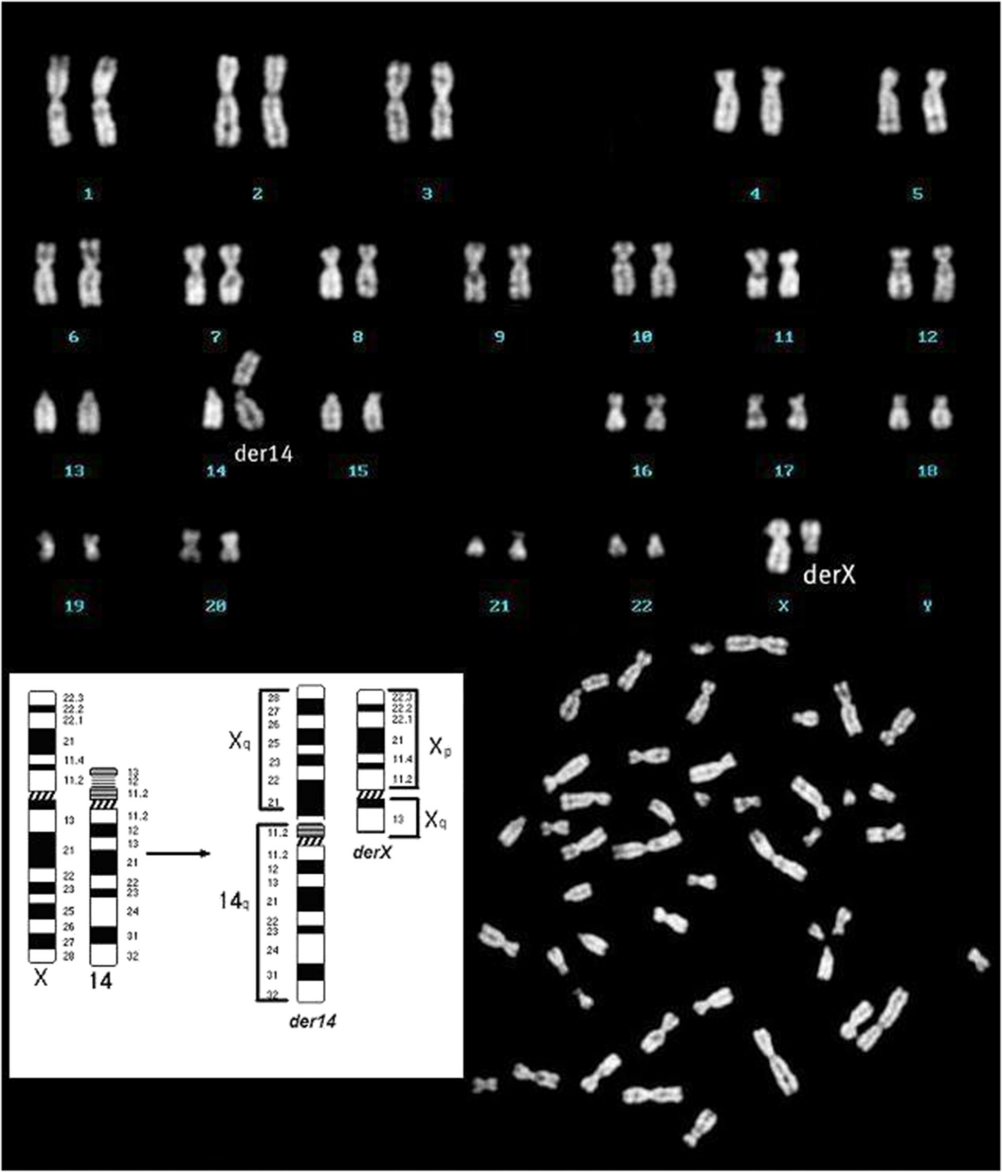
Karyotype 46, X, t(X; 14)(q13;p12).

It was necessary, at this point, to conclude the diagnostics by studying her karyotype. Her karyotype was obtained from T lymphocytes extracted from peripheral blood using the common culture technique. The obtained chromosomes were banded with Q-banding methods using quinacrine. An examination of her karyotype revealed the presence of a mutual translocation, apparently balanced, that involved the X chromosome long arm (q13) and the 14 chromosome short arm (p12), with consequent karyotype 46,X,t(X;14)(q13;p12) (Figure 1).

**Figure 2:**
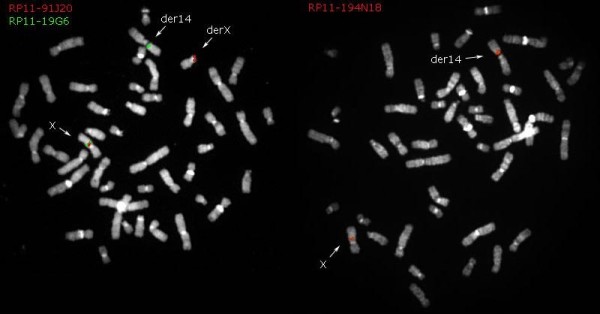
**BAC clones used for fluorescent *in situ *hybridization with the relative position on the X chromosome.** In the two images you see the signs of the clones which define the breakpoint on X. Also see Table 2.

Breakpoints were confirmed with fluorescent *in situ* hybridization (Figure 2, Table 2). To evaluate the inactivation of the X chromosome, we used the human androgen receptor (HUMARA) assay, which uses the locus of the androgen receptor in Xq11.2. In the first exon of the gene there was a highly polymorphic tri-nucleotide repetition (CAG) next to the cleavage sites of restriction enzymes sensitive to methylation (HpaII or HhaI), methylated only on the inactive X chromosome. The assay demonstrated that the active X chromosome was translocated (X-autosome: X; 14).

**Table 2:** **BAC clones used for fluorescent *in situ *** **hybridization with its location on the X chromosome**

**Clone**	**Band**	**Position**	**FISH resolution**
RP11-450P7	Xp11.1	chrX:21,533,785-21,657,970	derX
RP11-21E13	Xp11.1	chrX:57,294,374-57,500,705	derX
RP11-63004	Xq13.3	chrX:73,819,700-73,985,772	derX
RP11-91J20	Xq13.3	chrX:74,412,682-74,572,432	derX
RP11-194N18	Xq13.3	chrX:75,606,737-75,754,256	der14
RP11-28L16	Xq21.1	chrX:76,249,350-76,405,210	der14
RP11-19G6	Xq21.1	chrX:77,789,620-77,954,427	der14
RP11-91G23	Xq21.1	chrX:79,149,705-79,309,977	der14
RP11-336F4	Xq21.1	chrX:83,463,633-83,463,726	der14
RP11-210I11	Xq21.31	chrX:90,459,260-90,599,859	der14
RP11-138B3	Xq22.1	chrX:98,535,557-98,698,689	der14
RP11-265K3	Xq28	chrX:154,603,527-154,763,828	der14

The clones can be displayed on the site of the University of California, Santa Cruz [8]. FISH: fluorescent *in situ* hybridization.

To assess whether this was a *de novo* or a segregating chromosomal abnormality, we studied the karyotype of both parents. Their karyotypes were normal, implying that the chromosomal abnormality was *de novo*. Furthermore, our patient did not present clinical manifestations associated with X-linked recessive diseases.

## Discussion

X-autosome translocations are extremely rare (one in 30,000). In women, one of the X chromosomes is inactive, and this inactivation is completely random. This phenomenon is called lyonization. In cases of X-autosome translocation, the inactivation is not random but involves the none-translocated X chromosome [9,10].

We must consider that the X-inactivation center in the Xq13 area is turned off in the translocated chromosome, and different genes, which are important for the development and/or the functionality of the ovary, are present on the long arm of chromosome X [11].We can assume that X-autosome translocations do not interrupt the genes involved in ovarian functionality, but that they cause altered expression, because of their ‘position effect’ [12].

In females with an active X chromosome translocated in all cells and with the breakpoint not interrupting any functional gene, about half have ovarian failure (breakpoints within the Xq13 to q26 region) and the other half have a normal phenotype (breakpoints outside the Xq13 to q26 region) [13-15].

## Conclusions

The purpose of this therapy was not only to intervene in her climacteric symptoms, but to realize at the same time primary and/or secondary prevention of osteoporosis, cardiovascular pathology and cerebral involutional pathologies.

Our study has underlined that karyotyping is one of the fundamental investigations in the evaluation of amenorrhea. It has highlighted a genetic etiology for amenorrhea in the form of a chromosomal abnormality.

## Consent

Written informed consent was obtained from the patient for publication of this case report and accompanying images. A copy of the written consent is available for review by the Editor-in-Chief of this journal.

## Competing interests

The authors declare that they have no competing interests.

## Authors’ contributions

DD analyzed and interpreted the patient data and wrote the manuscript. AT, EM, GNM, AM and EP worked up the clinical details and helped to prepare the manuscript. AAE, DL and ML studied the androgen receptor gene. OC and MR performed the fluorescent *in situ* hybridization. All authors read and approved the final manuscript.
